# Hydrogel based scaffolds in cardiovascular disease: applications in myocardial regeneration, biological pacing, and heart failure therapy

**DOI:** 10.3389/fcell.2025.1678848

**Published:** 2025-10-21

**Authors:** Jiakun Tian, Huiling Yang, Linlin Chang, Xu Zhang, Weitie Wang, Ranwei Li

**Affiliations:** ^1^ Department of Intensive Care Unit of the Second Hospital of Jilin University, Changchun, Jilin, China; ^2^ Department of Anesthesiology and operating room of the Second Hospital of Jilin University, Changchun, Jilin, China; ^3^ Department of 2nd Gynecologic Oncology of Jilin Cancer Hospital, Changchun, Jilin, China; ^4^ Department of Cardiovascular of the Second Hospital of Jilin University, Changchun, Jilin, China; ^5^ Department of Urology of the Second Hospital of Jilin University, Changchun, Jilin, China

**Keywords:** hydrogels, cardiovascular diseases, myocardial infarction, biological pacing, myocardial regeneration, heart failure

## Abstract

Cardiovascular diseases are the leading cause of morbidity and mortality worldwide. Current treatments for cardiovascular diseases have improved overall survival rates. However, there are several limitations in reversing left ventricular hypertrophy, preventing ischemia-reperfusion injury, and completely curing refractory heart failure. Tissue engineering, such as the use of hybrid scaffolds, provides a promising strategy for solving these problems and is used in cardiovascular diseases, especially in the treatment of myocardial infarction. Hydrogels are materials with unique 3D crosslinked polymer networks. They are composed of natural or synthetic hydrophilic polymers that contain many chemical components. Hydrogels serve as effective materials in drug delivery and tissue regeneration, achieving desired therapeutic effects for a range of diseases. This review aims to introduce the types of tissue engineering materials currently used, including natural and synthetic hydrogels. In addition, this review investigates the application of hydrogels in cardiovascular diseases such as myocardial infarction, biological pacing, myocardial regeneration, and heart failure.

## 1 Introduction

As the foremost global killer, cardiovascular diseases claim more lives annually than any other cause of death (Martin et al., 2024). After myocardial ischemic infarction, necrotic myocardial tissue undergoes three stages: early inflammation, fibrosis, and long-term remodeling. During this period, the infarcted heart wall becomes thinner, and the left ventricle expands, ultimately leading to heart failure. Partial myocardial lesions can also cause changes in cardiac electrical conduction signals, leading to heart failure. Currently, the treatment of heart failure mainly includes three methods: medical therapy, left ventricular assist device implantation, and heart transplantation (Willerson, 2019). Drug therapy is suitable for early mild heart failure and should be actively administered for primary disease. While drugs are essential for managing early-stage heart failure and underlying conditions, often fail to halt or reverse the adverse ventricular remodeling that follows myocardial infarction, ultimately leading to pump function deterioration. For patients with advanced heart disease, mechanical circulatory support with left ventricular assist devices can be life-sustaining. However, the implantation of left ventricular assist devices is expensive and unsuitable for all patients with heart failure. Heart transplantation is the only effective treatment for end-stage heart failure, but its efficacy is limited by factors such as the limited availability of donors and immune rejection after transplantation. In addition, lifelong use of immunosuppressants can increase the risk of infection and malignant tumors (Shah et al., 2019). Therefore, there is an urgent need to develop new treatment methods to promote the repair of the damaged myocardium, thereby improving patient prognosis and quality of life.

Tissue engineering materials comprising scaffolds, cells, and growth factors have emerged as the most promising treatment strategies for cardiovascular tissue repair. To better repair cell damage, a scaffold (Table 1) should be biodegradable, simulate the characteristics of specific tissues, and provide the necessary cells or growth factors to support the newly formed tissues. Ideally, repair materials should have nontoxic and harmless properties, porous properties, biocompatibility, and the ability to differentiate cells and generate tissues. Simultaneously, the degradation rate of a perfect material should match the rate of new tissue formation. It should be able to transport nutrients and metabolites, tightly integrate with the surrounding natural tissues, and fill damaged areas (Balakrishnan and Banerjee, 2011).

**TABLE 1:** Hydrogel type and advantages.

Polymer	Sort	Advantages	Disadvantages
Alginate	Natural	1. Easy to acquire 2. Injectability 3. Calcium coagulation 4. Low cost 5. Hydration viscoelasticity, 6. Good biodegradability 7. Good biocompatibility	1. Poor stability 2. Poor adhesion 3. Poor mechanical propertie
Chitosan	Natural	1. Good biocompatibility, 2. Injectability 3. Acidic pH coagulation 4. Antioxidant 5. Antibacterial 6. Adsorption 7. Good biodegradability 8. Good biocompatibility	1. Poor mechanical performance 2. Extremely soluble
Gelatin	Natural	1. Good adhesion, 2. Injectability 3. Good biocompatibility, 4. Good degradability 5. UV coagulation	1. Poor thermal stability 2. Poor mechanical stability
Collagen	Natural	1. Similar to cartilage components 2. Injectability 3. Temperature coagulation 4. Good biocompatibility 5. Good adhesion	1. Poor mechanical performance 2. Degrade rapidly
PNIPAAm	Synthesis	1. Injectability 2. Temperature coagulation 3. Reversible Sol-gel Conversion 4. Shrinkage/swelling characteristics	1. Degradation 2. Slow thermal response rate 3. Poor mechanical performance
Graphene oxide nanoparticles	Synthesis	1. Good lubrication performance 2. Injectability 3. Temperature coagulation 4. Dispersion stability	1. Cytotoxicity 2. Genotoxicity
Cyclic peptide	Synthesis	1. Anti tumor 2. Antibacterial 3. Injectability 4. MMP coagulation	1. Dimerization into rings 2. High cost 3. Difficult to synthesize
PEG	Synthesis	1. Good biocompatibility 2. Hydrophilicity, 3. Injectability 4. Temperature coagulation 5. Anti protein adsorption 6. Non immunogenic	1. Fragile 2. Poor water swelling property

## 2 Classification of hydrogels

**FIGURE 1:**
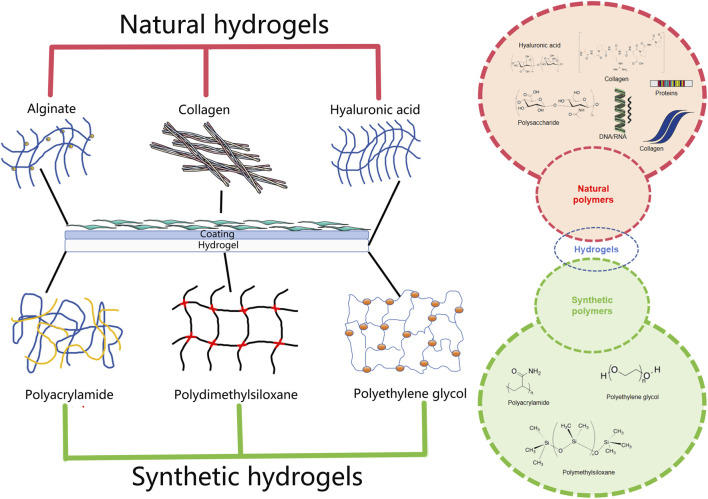
The chemical and physical network in hydrogels.

Natural polymer-based hydrogels typically exhibit good biocompatibility, low cytotoxicity, and similarity to physiological environments (Table 2). However, their disadvantages include weak mechanical properties, potential immunogenicity, uncontrolled degradation, and differences between various batches (Figure 1). Although natural polymer-based hydrogels have the above shortcomings, they are still promising biomaterials and have been proven to be suitable for heart tissue engineering (Hong et al., 2019; Mann et al., 2016). Currently, the natural polymers used in cardiac tissue engineering include alginate, chitosan, decellularized extracellular matrix (dECM), collagen, fibrin, gelatin, matrix gel, and hyaluronic acid. Below, we introduce several common sources for these materials.

**TABLE 2:** Classification of hydrogels.

Source	Nature	Alginate, Chitosan, Collagen, Fibrin, Gelatin
Composition	Synthetic	PNIPAAm, PEG
	Semi-synthetic	Two components materials
	Homopolymer	Monomer molecular composition with high crystallinity, high purity, and relatively low melting point
Crosslinking	Copolymer	Two or more different monomer molecular compositions
	Semi-IPN	An uncrosslinked linear molecule, intercalated in another crosslinked polymer
	IPN	Using chemical methods to interpenetrate two or more polymers into an interwoven network
	Physical junction	Hydrogen bonding, Amphiphilic graft and block polymers formation, Crystallization, ionicinteractions, Protein interactions
Hydrogels	Chemical binding	Chemical reaction, High energy radiation, Free radical polymerization, Enzymes
Configuration	Amorphous	A rigid solid with high hardness and high viscosity comparable to crystalline materials
	Crystalline	After cooling the hot saturated solution, solutes precipitate in the form of crystals
	Semi-Crystalline	It is a state of matter that distinguishes polymers from amorphous materials
Ionic Charge	Nonionic	
	Anionic	
	Cationic	
	Ampholytic	
Property	Mechanical strength	
	Biocompatibility	
	Biodegradability	
	Swelling	
Response	Physical response	Temperature, Electric field, Magnetic field, Light, Pressure, Sound, Humidity
	Chemical response	pH, ionicstrength, Solvent Composition, Molecular species, Redox reaction

### 2.1 Alginate hydrogel

Alginate is a natural anionic polysaccharide that is extracted from the cell walls of brown algae and certain bacterial capsules. β-D-mannuronic acid (M) and α-L-guluronic acid (G) are connected by (1→4) bonds. Owing to its good biocompatibility, high hydration viscoelasticity, biodegradability, cross-linking ability, physical properties, non-thrombotic properties, and low cost, it interacts with divalent cations (such as Ga^2+^, Ba^2+^, and Zn^2+^) to initiate gel formation, which is widely used in tissue engineering and has been approved by the Food and Drug Administration for medical treatment (Tritz et al., 2010; Cho et al., 2009). Compared with other natural polymers, alginate is conducive to cell function and anchoring, forming a 3D porous hydrogel scaffold (Zehnder et al., 2015). However, the application of alginate hydrogels has certain limitations. First, alginate hydrogels have poor stability and gradually lose their mechanical strength in a short time, even in a physiological environment, requiring additional substances during the design of the crosslinking process to enhance their mechanical properties (Vallée et al., 2009). Secondly, owing to the low cell adhesion of alginate in mammals, it is necessary to introduce cell-adhesion substances to better support cell function. For instance, cell-adhesive peptides containing the Arg-Gly-Asp sequence-a canonical ligand for integrin receptors-are frequently covalently grafted onto the alginate polymer backbone. In addition, the poor mechanical properties of alginate hydrogels limit their potential application in medicine. Finally, based on electrostatic and covalent interactions, the alginate-polymethacrylate hybrid hydrogel was used as the skeleton to prepare the scaffold material by cross-linking to form a single porous structure and to overcome the mechanical property limitations of the pure alginate material. Alginate hydrogels provide a suitable microenvironment to simulate the ECM, and methacrylate can improve the mechanical properties of mixed hydrogels (Stagnaro et al., 2018).

### 2.2 Chitosan hydrogel

Chitosan is derived from chitin, which is a cationic polymer from crustacean shells. It is a linear cationic polysaccharide with excellent hydrophilicity, biocompatibility, and biodegradability. Chitosan is an important component of connective tissue with chelating, antibacterial, adsorption, and antioxidant effects (Jayakumar et al., 2005; Oprenyeszk et al., 2015; Abd El-Hack et al., 2020). Chitosan is soluble in weak acidic solutions (pH < 6.5) where its amine groups become protonated, but it precipitates or dissolves poorly at physiological pH (7.4), limiting its direct use. For practical biomedical applications, chitosan hydrogels are designed to be stable under physiological conditions. Chitosan has good biocompatibility and degradability, and is considered a tissue engineering material with broad application prospects. Chitosan hydrogels can be produced by the N-deacetylation of chitin. A variety of external stimuli such as light and temperature can trigger a sol-gel reaction to form a 3D network structure (Shu et al., 2015).

A mixture of chitosan and other natural polymers can be used to generate a series of functional hydrogels via electrostatic interactions. However, the physically cross-linked chitosan network is extremely soluble, its mechanical properties are poor, and it has a certain sensitization, which greatly limits its application (Yang et al., 2018). At the same time, to overcome the defect of being insoluble in water, an N-succinyl chitosan dialdehyde starch mixture hydrogel with good solubility was invented (Kamoun, 2016). Compared with the pure chitosan hydrogel, the compressive modulus of the composite scaffold increased significantly after the introduction of the polycaprolactone scaffold, which was beneficial for cell survival. Therefore, composite hydrogel scaffolds often exhibit satisfactory mechanical strength and bionic microenvironments.

### 2.3 Gelatin hydrogel

Gelatin is a water-soluble protein produced by the partial hydrolysis of collagen. It is composed of arginine-glycine aspartic acid sequences and exhibits good biocompatibility, degradability, and temperature sensitivity. Its highest critical dissolution temperature is between 25 °C–35 °C, and it can improve cell adhesion and matrix metalloproteinase activity. Gelatin hydrogels have been used in tissue engineering and as molecular carriers in biomaterials. However, pure gelatin hydrogels exhibit poor thermal and mechanical stability. Therefore, it is necessary to chemically modify the structure of gelatin to improve its physical and chemical properties and make it more suitable for application in the field of cardiovascular repair. The modification of gelatin hydrogels with methacryloyl anhydride to obtain gelatin methacryloyl (GelMA) is a common and effective modification method (Datta et al., 2017). The GelMA hydrogel exhibited the highest water absorption rate and equilibrium swelling ratio under UV light irradiation. Typically, a photoinitiator is used at concentrations of 0.1%–0.5% (w/v), and crosslinking is induced under UV light (365 nm wavelength) at an intensity of 5–10 mW/cm^2^ for periods of 30–120 s. These parameters allow for precise control over the crosslinking density, which directly dictates the hydrogel’s mechanical modulus (1–20 kPa for cardiac applications) and degradation profile. The degradation speed was fast, the tensile properties were similar to those of the natural tissue structure, and the material had good biocompatibility (Daniele et al., 2014). The chemical modification of gelatin to prepare biological scaffolds provides a new approach for the treatment of cardiovascular diseases.

### 2.4 Collagen hydrogel

Collagen is an important component of the ECM in mammalian cells and is a natural biomaterial composed mainly of type I and type II collagen. It has a triple-helical structure and is widely present in skin, bones, cartilage, blood vessels, teeth, and tendons. It is widely used in biology and medicine. Collagen is an important component that maintains the structural and functional integrity of the heart, provides mechanical support and tensile strength, and is connected to myocardial cells through integrins. Collagen hydrogels can be prepared by UV photopolymerization, dehydrogenation, heat treatment, or cross-linking with other groups. The properties of the composite hydrogels prepared from type I and type II collagen can be adjusted by changing the content of the two collagen types. Collagen is usually combined with other natural biological macromolecules or is chemically modified to form hydrogels. Injectable hydrogels prepared using type II collagen and hyaluronic acid can maintain the activity and phenotype of chondrocytes in the process of culture (Kontturi et al., 2014). Similar to gelatin, collagen has poor mechanical properties and can rapidly degrade, which is detrimental to tissue repair. The improvement in collagen was similar to that of gelatin. Recently, collagen hydrogels have developed rapidly in the field of heart tissue engineering. Currently, biotechnology-produced collagen avoids the risk of allergic reactions and pathogen transmission associated with animal-derived collagen.

### 2.5 dECM

**FIGURE 2:**
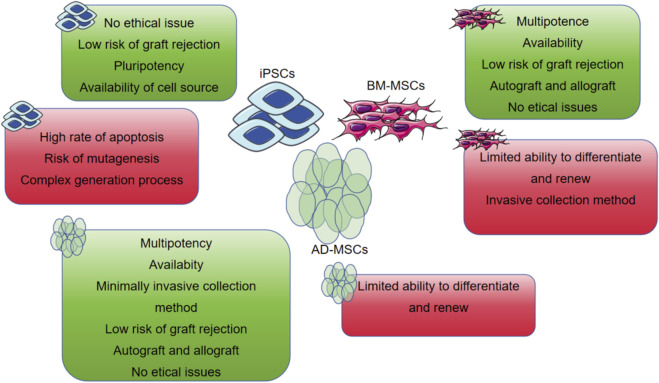
The most common used MSCs encapsulated in the hydrogel with their own advantages and disadvantages.

dECM is obtained by decellularizing tissues, retaining the microstructure and bioactive components of natural ECM (such as proteoglycans and glycosaminoglycans), simulating the cellular microenvironment, and possessing advantages such as biological activity, biocompatibility, and degradability (Datta et al., 2017). Human cardiomyocytes cultured in cardiac dECM hydrogels exhibit significantly increased expression of cardiac-specific markers like Troponin T and Connexin 43, alongside improved synchronous beating behavior, compared to those in plain collagen or synthetic hydrogels. The 3D structure and biochemical composition of the ECM are specific to each tissue. In cardiac repair, myocardial ECM materials can support cell survival and *in vivo* biological behavior (Datta et al., 2017). Human bone marrow mesenchymal stem cells (hMSCs) inoculated on porcine myocardial ECM hydrogels show consistent arrangement and growth characteristics with human induced pluripotent stem cell-differentiated cardiomyocytes (hiPS-CMs) and form an organizational structure (Efraim et al., 2019) (Figure 2). Generally, ECM hydrogels have low mechanical strength, slow gelation processes, and fast degradation rates. Therefore, some researchers have added genipin crosslinkers or matrix metalloproteinase inhibitors (such as doxycycline) during the preparation of ECM hydrogels to slow their degradation rate.

### 2.6 Fibrin hydrogel

Fibrin is a natural polymer that is the product of physiological coagulation reactions and participates in hemostasis and wound healing (Heher et al., 2018). Fibrin hydrogels are produced by a mixed reaction between fibrinogen and thrombin. Thrombin splits and removes fibrin peptides A and B from fibrinogen and exposes the polymerization site of the fibrin monomer; these monomers then self-assemble into a hydrogel network (Heher et al., 2018). Fibrin hydrogels exhibit good biocompatibility, low immunogenicity, and controllable degradation rates. In addition, fibrin hydrogels can promote cell adhesion and uniform distribution and improve the survival rate of inoculated cells.

### 2.7 Matrigel

Matrigel is an ECM protein mixture extracted from Engelbreth-Holm swarm mouse sarcoma and is mainly composed of type IV collagen, laminin, and heparin sulfate glycoprotein (Kastana et al., 2019). Matrigel is commonly used as a substrate coating to support the adhesion and growth of various cells and is currently the preferred substrate for stem cell culture. When the concentration of Matrigel is greater than 4 mg/mL, it can gel at 24 °C–37 °C. However, because of the possibility of introducing heterologous contamination and tumorigenicity issues, Matrigel has not yet been approved for clinical research. Efforts in the field are increasingly focused on developing defined, synthetic, or human-derived hydrogel alternatives to recapture Matrigel’s bioactivity in a clinically compliant manner.

Synthetic hydrogels exhibit easily adjustable mechanical and biochemical characteristics. Their mechanical strength, pore size, gelation time, and degradation rate can be controlled according to the application of the gel. In addition, they exhibit low immunogenicity and batch consistency. Synthetic hydrogels compensate for some deficiencies of natural hydrogels and have good application prospects in heart repair. Several commonly used synthetic hydrogels are discussed below.

### 2.8 Poly N-isopropylacrylamide hydrogel

Poly-N-isopropylacrylamide (PNIPAAm) is a thermosensitive polymer with thermoreversible gelatin characteristics. When the temperature of this polymer is higher than its critical solution temperature (32 °C), it can undergo sol-gel transformation (Nagase, 2021). When PNIPAAm is injected into the body, it can be used as an in-situ gel; however, the poor biodegradability of the hydrogel limits its direct application. To design a PNIPAAm hydrogel with good biodegradability and biocompatibility, researchers have introduced biodegradable and/or natural polymers into its molecular structure.

### 2.9 Polyethylene glycol hydrogel

Polyethylene glycol (PEG) is a synthetic hydrogel widely used in tissue engineering (Chu et al., 2023). It has good biocompatibility, low immunogenicity, and low toxicity, and has been approved by the FDA for use in the medical industry. Although PEG is biologically inert and non-degradable *in vivo* with low adhesion to cells, which is not conducive to cell survival and growth, it can be easily conjugated with various polymers to overcome these problems. PEG and its modified hydrogels have been used for heart repair after myocardial infarction (MI).

### 2.10 Self-assembled peptide hydrogel

Self-assembled peptide hydrogels can imitate the natural ECM with good biocompatibility, degradability, no immunogenicity, and cytotoxicity, making them promising biomaterials. When designing self-assembled peptide hydrogels, different secondary and tertiary structures can be constructed by adjusting the amino acid sequences. The implementation of tertiary structures and the introduction of bioactive groups can functionalize peptides (Luo et al., 2022). Self-assembled peptide hydrogels can be used as carriers for the delivery of cells, biomolecules, and drugs. Sol-gel transitions can occur under the influence of temperature, pH, or ionic strength.

### 2.11 Mixed hydrogel

Natural and synthetic hydrogels exhibit unique characteristics. Natural hydrogels have good biocompatibility and biological functions; however, they have shortcomings such as weak mechanical strength, slow gel speed, and differences between various batches. Synthetic hydrogels have mechanical strength and physicochemical properties that are easy to control but lack cell adhesion sites. Therefore, to optimize the performance of hydrogels, a promising method is to selectively combine natural and synthetic materials to prepare mixed hydrogels according to their characteristics. The designed mixed hydrogel has cell adhesion sites, appropriate mechanical strength, and physical and chemical properties that meet the requirements for heart repair (Jin and Stanciulescu, 2017).

At present, the number of tissue engineering products used in clinical trials is limited, and ideal hydrogels should have the following characteristics at the same time: ① Low immunogenicity, biological activity and bionic function; ② Endures mechanical stress; ③ Good biocompatibility, combines with cells and vascular tissues; and ④ Transports drugs and growth factors. Therefore, the development of composite hydrogel scaffolds with complex structures modified by various components is necessary to achieve good clinical therapeutic effects; corresponding research on the mechanical and biological behavior of the hydrogel is carried out to ensure strong interactions between materials and surrounding tissues. In the future, we should design secondary and tertiary advanced hydrogel structures; quantify their composition, morphology, structure, and function; characterize their binding modes and interactions with cell surface receptors; and transform them into clinically usable biomaterials in cardiac tissue engineering.

Beyond traditional classifications, we propose a novel functional taxonomy tailored for cardiovascular applications (Table 3), categorizing hydrogels based on their primary therapeutic objective: ① Structural Support Hydrogels: Primarily aimed at providing mechanical support to the infarcted wall to prevent adverse ventricular remodeling (e.g., Algisyl-LVR, IK-5001). ② Conductive/Bio-pacing Hydrogels: Engineered to restore electrical conduction in scarred myocardium or host pacemaker cells (e.g., polypyrrole-chitosan composites, PAMB-G). ③ Drug/Cell Delivery Hydrogels: Designed as controlled-release systems for therapeutic agents (e.g., growth factors, miRNAs, exosomes) or as cell carriers to enhance retention and survival. ④ Smart Responsive Hydrogels: “Intelligent” materials that respond to specific pathological microenvironments (e.g., pH, enzyme levels) to activate therapy on demand (Table 4).

**TABLE 3:** Biomedical applications of protein-based hydrogels.

Function	Hydrogels types	Advantages	Mechanism
Bioadhesion	1. Pectin or pectin-containing gels	Forms strong, biocompatible interfaces with target tissues	Pectin chains interact with biological surfaces to form cohesive bonds
Drug delivery	1. Poly(D,L-lactide-co-glycolide) (PLGA)/PEG triblock copolymers (PLGA-PEG-PLGA) 2. Alginate/PEG/hyaluronic acid 3. RADA16 4. Nap-GFFYGGGWRESAI/TIP crosslinker 5. Leucine-α/β-dehydrophenylalanine	1. Tunable degradation profile for controlled release kinetics 2. Responsive to environmental stimuli (e.g., pH, temperature)	Engineered to exhibit complex release kinetics for their associated therapeutic payloads
Wound healing	1.PEG-diacrylate (PEGDA) 2. Acrylic acid 3. Alginate ionic concentration driven	1. Provides a moist, protective barrier 2. Can encapsulate and release antimicrobials or growth factors 3. Reduces inflammation and supports tissue homeostasis	1. Creates a stable scaffold that supports cell migration and tissue ingrowth. 2. Sustained delivery of bioactive molecules promotes healing
3D cell culture	1. Fibrin 2. Alginate 3. PEG	1. Biochemically and mechanically tunable to mimic native ECM. 2. Supports cell adhesion, proliferation, and 3D organization	1. Provides a three-dimensional porous scaffold that allows for nutrient/waste diffusion and cell growth in all dimensions
Tissue regeneration	1. CollagenKeratin–fibrinogen 2. Collagen 3. PEG	1. Excellent biocompatibility and bioactivity 2. Attracts cell migration and promotes differentiation	Acts as a temporary ECM analog, providing mechanical support and biochemical cues to guide new tissue formation
Biofabrication	1. Alginate/fibrinogen 2. GelMA 3. PEO	1. Printable and moldable for creating complex structures 2. Can be modified to fine-tune cellular interactions	Combines the advantages of different materials to create optimized bio-inks for 3D printing or patterning of tissues
Wearable sensor	1. Wristbands 2. Strip-type sensors	1. High elasticity and conformability to skin 2. Can be functionalized with chemical or electronic sensing elements	Serves as a flexible, water-rich matrix that houses sensing components and facilitates signal transduction from the body to the detector

**TABLE 4:** Characteristics of natural versus synthetic hydrogels.

Property	Natural hydrogels	Synthetic hydrogels	Remarks/Clinical implications
Source & Composition	Derived from biological sources	Chemically synthesized from defined monomers	Natural hydrogels may carry risks of batch-to-batch variation and immunogenicity; synthetic hydrogels offer superior reproducibility
Biocompatibility & Bioactivity	Typically excellent; often contain innate cell-adhesion motifs	Generally good, but often bio-inert unless functionalized	Natural hydrogels inherently promote cell adhesion and interaction; synthetic hydrogels require modification to enhance bioactivity
Mechanical Properties	Relatively soft and weak; limited and difficult to tune independently	Highly tunable and reproducible over a wide range	Synthetic hydrogels can be engineered to better match the mechanical properties of native heart tissue
Degradation Time	Often fast and enzymatically controlled; can be unpredictable	Tunable and predictable, primarily through hydrolysis	Controlled degradation is critical to match the rate of new tissue formation. Synthetic hydrogels offer better control
Delivery Methods	Primarily injectable	Injectable or pre-formed scaffolds	Injectable hydrogels enable minimally invasive delivery via catheter, which is highly suitable for cardiac applications
Key Advantages	1. Inherent bioactivity 2. High, biocompatibility 3. Natural cell interactions	1. Precise control over properties 2. Excellent reproducibility 3. High mechanical strength	The choice depends on the application: natural for bioactivity, synthetic for structural control. Hybrid hydrogels aim to combine both advantages
Major Limitations	1. Batch-to-batch variation 2. Poor mechanical strength 3. Uncontrolled degradation	1. Lack of inherent bioactivity 2. Potential toxicity of degradation products	1. Lack of inherent bioactivity 2. Potential toxicity of degradation products 3. Natural hydrogel limitations relate to their source, while synthetic hydrogel limitations relate to their biological integration

## 3 Design criteria for cardiac scaffolds

### 3.1 Biological characteristics

**FIGURE 3:**
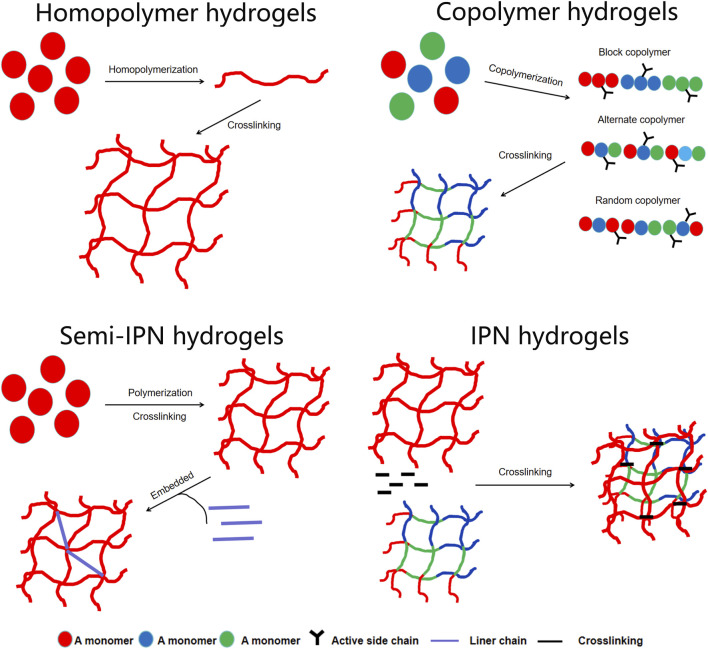
The polymers in the hydrogels can be homopolymers, copolymers, semi-interpenetrating network (semi-IPNs), or IPN hydrogels.

Hydrogels are materials with unique 3D crosslinked polymer networks. They are composed of natural or synthetic hydrophilic polymers that contain many chemical components and are expressed through their characteristics (Chen et al., 2023). Polymers in hydrogels can be homopolymers, copolymers, semi-interpenetrating networks (IPN), or IPN hydrogels, depending on their composition (Figure 3). Hydrogels consist of a type of polymer material that uses a liquid as a dispersing medium with hydrophobic and hydrophilic groups to form a polymer chain. The hydrophilicity of the polymer chain enables it to absorb a certain amount of water. Thus, the hydrogel can maintain its 3D network structure while absorbing a large amount of water due to the cross-linked polymer chains, whereas the hydrophilic groups within the network are responsible for the water uptake and swelling behavior (Liang et al., 2023), which enables drug delivery and tissue regeneration. Therefore, hydrogels can not only absorb a large amount of water but also maintain a 3D network structure without dissolving in water, which is conducive to the diffusion and exchange of oxygen, nutrients, and metabolites. It functions in water absorption, retention, and slow release, similar to the extracellular matrix (ECM) in the organism. In addition, hydrogels can provide a 3D growth environment for cells and mechanical support to surrounding tissues (Kamimura et al., 2014).

**FIGURE 4:**
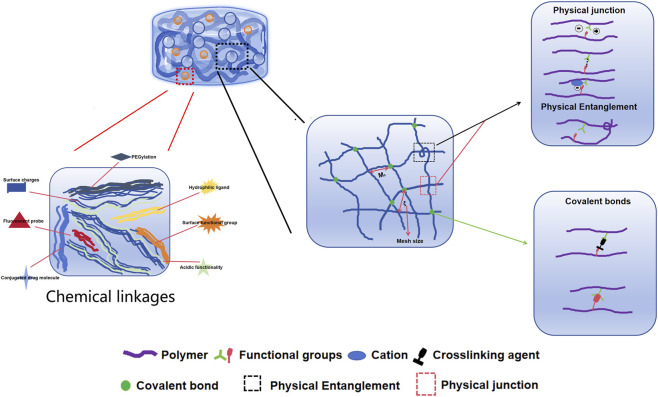
Injectable natural hydrogels (formed from alginate, gelatin and hyaluronic acid) and synthetic hydrogels (formed from polyacrylamide, polydimethylsiloxane and polyethylene glycol) commonly used as substrates for cardiovascular research.

Based on their cross-linking properties, hydrogels can be divided into chemical and physical network gels (Figure 4). Chemically crosslinked hydrogels are usually bound by the molecular bonds of synthetic polymers and are characterized by their stability, identical composition, and controllable structure. For instance, PEG diacrylate hydrogels, a benchmark synthetic system, can achieve G′ values in the range of 1–10 kPa, making them suitable for providing mechanical support to the infarcted wall. Physically crosslinked hydrogels are usually aggregated by secondary interactions such as molecular entanglement, hydrogen bonds, ionic bonds, or hydrophobic interactions. For example, a thermosensitive chitosan/β-glycerophosphate hydrogel transitions from a liquid (G”' > G’) at room temperature to a solid-like gel (G’ > G”) at body temperature. The structure is reversible and exhibits self-healing characteristics. Compared to chemically crosslinked hydrogels, physically crosslinked hydrogels have many advantages, such as easy manufacturing, low toxicity, remolding, and biodegradability (Eslahi et al., 2016). According to their response to external stimuli, hydrogels can be divided into traditional and environmentally sensitive hydrogels. Based on their properties, hydrogels can be divided into neutral and ionic hydrogels. They can be divided into natural and synthetic hydrogels according to their composition. Natural hydrogels are composed of hydrophilic polymers from natural sources such as cellulose, alginate, hyaluronic acid, chitosan, collagen, and poly-L-lysine. Synthetic hydrogels are composed of hydrophilic polymers such as polyacrylic acid, polymethacrylic acid, polyethylene oxide, polyethylene glycol, and polyvinyl alcohol (Ahmed, 2015). In the field of cardiac regenerative medicine, commonly used hydrogels include alginate, chitosan, ECM, self-assembled peptide, and fibrin hydrogels, which are used as carriers, matrices and scaffolds (Alagarsamy et al., 2019; Li et al., 2022; Saludas et al., 2017). Owing to their biocompatibility, degradability, and similarity to the ECM, natural hydrogels have a wide range of applications in tissue regeneration, providing cells with biological activity and a good adhesion surface. Natural polymers used to prepare hydrogels include protein-based materials (gelatin, collagen, fibrin, and silk protein) and polysaccharide-based materials (hyaluronic acid, chondroitin sulfate, alginate, and chitosan) (Hu et al., 2021). Natural hydrogels are non-toxic and do not cause immune reactions; their degradation products are non-toxic and non-immunogenic, and the final metabolites are safely excreted from the body. However, the poor stability, rapid degradation, and relatively low mechanical strength of natural hydrogels significantly limit their applications (Nair and Laurencin, 2007). Synthetic hydrogels have mechanical strength and physicochemical properties that are easy to control but lack cell adhesion sites.

The pore size between polymer molecules in the hydrogel network can range from 5 nm to 500 um. These pores are designed to induce tissue regeneration (Li and Mooney, 2016). Scanning electron microscopy (SEM) and transmission electron microscopy (TEM) are typically used to observe the surface morphology and internal porous structures of hydrogels and other biomaterials. The obvious difference between the two devices is the difference in spatial resolution. For example, SEM shows that hydrogels have a porous microstructure and smooth pore walls. In contrast, the internal structure of the hydrogel can be observed through TEM, and its resolution can reach 0.2 nm (Cao et al., 2014).

The physical properties of hydrogels are considered one of the most important factors controlling cell regeneration. These properties include their water-retention capacity, swelling capacity, and embedding rate. Their entrapment efficiency can be determined by spectrophotometry or high-performance liquid chromatography according to the different properties of the embedded drugs. Mechanical properties, such as the compressive modulus, tensile strength, and rheological properties, are important indicators for evaluating hydrogels made from different materials and preparation methods. Because the presence of different functional groups and chemical bonds has a significant impact on the performance of hydrogels, it is necessary to analyze the different functional groups and chemical bonds in the prepared hydrogels. The structures of hydrogels are typically observed using X-ray diffraction, Fourier transform infrared spectroscopy, nuclear magnetic resonance, and thermogravimetry. When the hydrogel degradation test is conducted *in vitro*, the hydrogel material can be put into phosphate buffer saline and incubated at 37 °C for a specified time. As hydrogel materials can be used as scaffolds for cells, it is necessary to test their safety for embedded cells and implantation sites. *In vitro* biocompatibility tests are generally conducted through cell experiments, and the survival of cells in hydrogel materials is observed using a series of indicators. The morphology of cells in the hydrogel scaffold can be observed using an inverted microscopy mirror. Cell growth and proliferation can be determined by MTT assay, AO/PI staining and DAPI staining. Cell growth can also be evaluated by analyzing ECM synthesis, which can be stained with toluidine blue dye and observed under an inverted microscope (Ribeiro et al., 2018).

**FIGURE 5:**
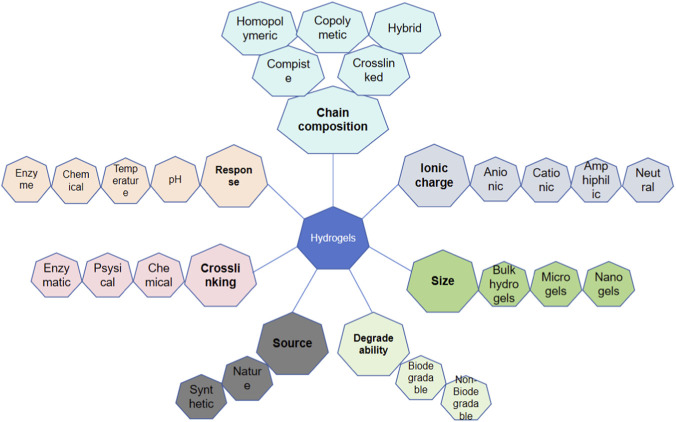
The basic characteristic of hydrogels including chain composition, response, crosslinking, source, degrade ability, size and ionic charge.

Injectable hydrogels are important components of heart tissue engineering. Injectable hydrogels have good biocompatibility, low immunogenicity, high permeability, and good mechanical properties (Figure 5). These hydrogels can also be infused into the myocardium through a catheter as a minimally invasive treatment. Hydrogels can be composed of a variety of natural or synthetically derived polymers and can be assembled into a 3D polymer network with high water content. These characteristics enable hydrogels to imitate the ECM environment as carriers for cell transplantation; promote cell survival, proliferation, differentiation, and migration; and promote tissue regeneration (Soltani et al., 2022; Giobbe et al., 2019; McLaughlin et al., 2019). Triggered by specific physical and chemical environments, hydrogels can undergo *in situ* sol-gel transformations, which usually involve thermal stimulation, pH mediated cross-linking, photoinduced cross-linking, ionic cross-linking, and chemical cross-linking. Among these, hydrogels based on thermal stimulation cross-linking are the most widely used. Thermosensitive hydrogels can undergo gel transformation under certain temperature conditions; therefore, injection into the myocardium can trigger *in situ* gel formation. In addition, hydrogels can be used to deliver biomolecules, drugs, and therapeutic genes to damaged myocardial tissues; release molecular control to target areas; improve the microenvironment of infarct areas; and promote endogenous cell recruitment and angiogenesis (Youngblood et al., 2018). In recent years, researchers have developed a variety of injectable hydrogels, which can be divided into natural polymer-based, synthetic, and mixed hydrogels. Different biomaterials have unique characteristics that should be fully considered before designing and manufacturing hydrogels, particularly in heart tissue engineering.

Tissue-engineered scaffolds are used for the physical support of cellular components in biotechnology. Therefore, the composition of biological scaffolds should be similar to that of the ECM of the heart to reduce myocardial tissue rejection after implantation (El-Husseiny et al., 2023). As a supporting structure for cellular components, tissue-engineered scaffolds should have sufficient pores to facilitate endothelial cell growth and the formation of new capillaries while allowing oxygen and nutrients in the blood to diffuse into the tissue structure through the pores. This would provide energy for implanted cells and new tissue structures to maintain their survival and promote their proliferation. In addition, certain types of tissue scaffolds provide the space and physical attachment required for the short-term growth of implanted cells. When implanted cells survive or proliferate, the tissue-engineered scaffold must be degraded for excretion from the body. Therefore, biological scaffolds also need to have self-degradation ability, and the degradation products should not be toxic to the body.

### 3.2 Cardiac tissue-engineering materials and platforms

#### 3.2.1 Hydrogel

Hydrogels have flexibility similar to that of heart tissue. Their high water content and good physical and chemical properties make them suitable for tissue engineering applications (Dhingra et al., 2014). In heart tissue engineering, hydrogels are typically used to inoculate the encapsulated cells and compress them into 3D tissues. Hydrogels promote cell survival and syncytial body formation, similar to the ECM. Moreover, the physiological characteristics of hydrogels can be integrated in different application situations so that they can better match the various environments (Duan et al., 2011). Hydrogels contain natural polymers, such as alginate, collagen, or fibrin. Sodium alginate hydrogels are commonly used for drug delivery and can help cells combine with drugs in a long-term manner. Sodium alginate hydrogels can capture cells and release drugs to combine via ionic or photo-crosslinking (Tandon et al., 2013). Other hydrogels can be processed and synthesized using synthetic polymers such as polyvinyl alcohol, polyethylene glycol, and polyacrylic acid (Hasan et al., 2015). Currently, several advanced hydrogels are used in heart therapy. For example, the angiopoietin one derived peptide—QHREDGS fixed on a chitosan collagen hydrogel—can promote the maintenance of cardiac function after MI (Reis et al., 2015). Compared to the control group, this modified hydrogel significantly improved cardiac function and reduced cardiac remodeling in a MI model in rats. The application of injectable hydrogels in cardiac tissue engineering has received extensive attention from researchers. An injectable hydrogel is prepared from a mixed liquid and can be delivered to the human body through a catheter, thereby avoiding the surgical procedure required for stent delivery. At the same time, injectable hydrogels can also be used to transfer cells and protein substances, such as growth factors, and can be added in various combinations to be manufactured and utilized (Pavo et al., 2014). The main disadvantage of injectable hydrogels is the lack of control over the final transplant site and number of cells. Riboflavin-5 phosphate is used to photosensitize the hydrogel, and UV-mediated surface light ablation of the hydrogel is used to achieve rapid and automated construction of custom organ chips, which has shown robustness and repeatability without changing the mechanical properties of the hydrogel. Rat or human cardiomyocytes are inoculated into this heart chip, and the resulting heart tissue acquires contractile function and long-term viability, which can maintain continuous cultivation for 27 days, allowing for long-term research on the chip and reducing the time by 60% compared with traditional methods. This process provides important technical support for the automation and continuous manufacturing of organ chips (Nawroth et al., 2018).

#### 3.2.2 Cell slice

A cell slice is a new scaffold-free technology that addresses the lower cell concentration in scaffolds compared to natural hearts and avoids the risk of inflammatory reactions caused by scaffold degradation. The production of cell slices begins with the cell culture and progresses to cell fusion. The surface of the cell culture dish is coated with a temperature sensitive polymer known as poly-N-isopropylacrylamide, which has hydrophobicity and adheres to cells at 37 °C. However, when the temperature drops to 32 °C, it causes the monolayer of cultured cells, along with the ECM and attachment proteins, to separate as a complete cell layer. When 2D cell layers accumulate, they can quickly form connections between cells, thereby preserving the ECM and intercellular connections between adjacent myocardial cells (Alrefai et al., 2015). After transplantation, the action potential of cell slices can improve cardiac function, vascular distribution, and fibrosis (Dilley and Morrison, 2014). The degree of vascularization increased when the ECs were sandwiched between multiple layers of myocardial cell sheets. Similarly, implanting MSC slices into the hearts of infarcted rats improved cell survival and left ventricular function and reduced ventricular dilation. Although cell slices are an attractive option, their tissue structures are fragile and difficult to use in clinical applications. At the same time, the thickness limit of the layered myocardial cell plate is about 80 um. When this thickness is exceeded, the vascular network formed cannot provide sufficient nutrition or oxygen to the tissue.

#### 3.2.3 Cardiac chip

A cardiac chip is a physiological microfluidic human heart model based on the principles and technologies of cardiac tissue engineering that is used for the development of cardiac drugs. Cardiac myocytes derived from human induced pluripotent stem cells are cultured in hydrogel components compressed with polydimethylsiloxane to construct a fibrous cardiac tissue microarray that can repeatedly produce cardiac myocyte fibers derived from human induced pluripotent stem cells. In addition, human-induced pluripotent stem cell-derived myocardial cell fibers can change their contraction frequency and peak contraction force according to the applied drug, and the changes in contraction properties are consistent with the efficacy of the drug when applied to the human body. Therefore, human-induced pluripotent stem cell-derived myocardial cell fibers can be used as an effective tool for drug development to replace human testing (Morimoto et al., 2016).

## 4 Applications in myocardial infarction and regeneration

### 4.1 Myocardial infarction

**FIGURE 6:**
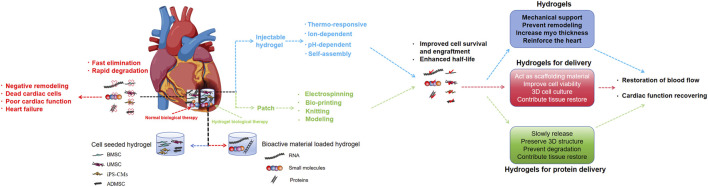
Hydrogels can be used as acellular scaffolds for cell and protein, which can improve the limitations of current cell and protein therapies for myocardial infarction.

Cardiovascular disease has a high incidence rate and mortality worldwide, and seriously affects health and quality of life. In the population aged 45-64 in the United States, the incidence of heart failure 5 years after the first MI is 8% in male patients and 18% in female patients (Mozaffarian et al., 2015) (Figure 6). MI is a common type of cardiopathy, and acute large-area MI can lead to sudden death. Small-area MI can also seriously affect long-term survival and quality of life. This is because it is difficult to effectively suppress negative ventricular remodeling after MI using current clinical treatment strategies. After MI, the heart undergoes a series of structural and functional changes such as myocardial cell necrosis, fibroblast proliferation and repair of necrotic areas, thinning of ventricular walls, enlargement of cardiac chambers, abnormal cardiac contraction and diastolic function, and reduced cardiac pumping function, which affect long-term survival and quality of life. After MI, myocardial cells undergo necrosis and cardiac fibroblasts proliferate to repair the necrotic area, forming scar tissue. Multiple factors work together to affect cardiac repair (Nishida and OTSU, 2016). Myocardial cell necrosis, injury, and fibroblast proliferation alter the normal heart structure, leading to cardiac dysfunction or heart failure. For patients with end-stage heart failure, heart transplantation is still a practical and effective treatment method; however, there are problems such as the limited number of donor organs and immune rejection in heart transplantation (Dogan et al., 2017). Therefore, there is an urgent need to identify novel therapeutic techniques that promote myocardial cell proliferation.

Various biomaterials have been developed with a 3D cross-linked polymer network structure that can mimic the ECM to facilitate cell adhesion and material exchange and that have appropriate mechanical strength to withstand ventricular stress (Pina et al., 2019). As an implant material for MI treatment, hydrogels have the following advantages: (1) they can provide mechanical support for weakly damaged myocardial tissue, promote cell adhesion, reduce scar tissue, and improve cardiac function; (2) as an ECM analog, they can sustainably deliver biological factors locally and accelerate angiogenesis; and (3) the use of injection reduces patient trauma and surgical complications. For patients with MI, intramyocardial injection of hydrogels can increase myocardial thickness and cause mechanical stress, supporting the left ventricular wall and delaying left ventricular remodeling (Rufaihah et al., 2017). Injectable hydrogels are a type of hydrogel that can exist in liquid and be transported to the injection site, respond to certain stimuli such as temperature and ionic strength, and rapidly complete the liquid-solid transition. They were first used for the treatment of MI in 2003 (Iwakura et al., 2003). From the initial exploration of MI models of small animals, such as rats and mice, to current clinical trials, MI models have developed at an astonishing rate. Some studies have shown that injecting hydrogels into myocardial tissue can increase wall thickness, inhibit ECM degradation, and provide temporary mechanical support. Simultaneously, it can reduce the inner diameter of the heart cavity, alleviate wall tension, lower myocardial oxygen consumption (MVO2), and delay the progression of heart failure. Foreign clinical studies have evaluated the feasibility and safety of three injectable hydrogels: Algisyl LVR (Mann et al., 2016), IK-5001 (Frey et al., 2014), and VentriGel (Traverse et al., 2019). Traverse et al. conducted a clinical study on the treatment of left ventricular dysfunction with VentriGel, which preliminarily confirmed the safety and feasibility of VentriGel injection and provided support for further large-scale clinical trials (Traverse et al., 2019). Research on injectable hydrogels and cardiac function repair has focused on enhancing hydrogel structure and function, such as their conductivity, antioxidants, and more complex chemical composition modifications.

Algisyl LVR and IK-5001 are mainly composed of alginate and were the first hydrogels to enter clinical trials (Frey et al., 2014). A Phase I clinical trial of Algisyl LVR (NCT00847964) included three patients with ischemic cardiomyopathy who received an intramuscular injection of Algisyl LVR during coronary artery bypass grafting. Six months later, magnetic resonance imaging of the heart revealed a decrease in left ventricular end-diastolic and end-systolic volumes and an increase in stroke volume, left ventricular ejection fraction, and wall thickness. Computer model simulation results showed a significant decrease in wall stress and stress anisotropy on myocardial fibers in patients. The Phase II clinical AUGMENT-HF trial (NCT01311791) confirmed that Algisyl LVR implantation in the myocardium of heart failure patients is safe and feasible and can effectively improve patients' exercise tolerance and heart failure symptoms, as well as cardiac function grading (Mann et al., 2016). However, this study did not meet its primary efficacy endpoints, indicating the need for further investigation into its therapeutic benefits. IK-5001, developed by Israeli scholars, can penetrate an infarcted area through damaged coronary arteries, forming a gel in the high-calcium environment of the infarcted area. However, it cannot pass through normal blood vessels or form a gel in the non-ischemic myocardium with a normal calcium ion concentration. During MI repair, IK-5001 is gradually degraded and replaced by myofibroblasts and connective tissue (Frey et al., 2014). A Phase I clinical trial (NCT00557531) confirmed that the treatment of ST-segment elevation MI with IK-5001 via coronary artery injection is safe and feasible without affecting coronary blood flow and myocardial perfusion. After a follow-up of 6 months, the patient’s heart function remained at preoperative levels, the heart cavity did not further expand, and the N-terminal pro-B-type natriuretic peptide (NT pro BNP) gradually decreased.

Although injectable hydrogels have entered the clinical trial stage for the treatment of MI, the exact mechanism is not yet fully understood, and may be related to the following mechanisms: Promote neovascularization and blood flow perfusion in the infarcted area; Promote stem cell homing and myocardial repair; Replace ECM, improve the microenvironment. According to Laplace’s law, a hydrogel injected locally into the infarcted myocardium can increase the thickness of the infarcted wall, reshape the ventricular geometry, and reduce the stress on the ventricular wall. Simultaneously, owing to the supporting effect of the hydrogel, the contradictory movement of the infarcted region weakens or disappears, and the ineffective work of the heart decreases, thus delaying negative ventricular remodeling and protecting cardiac function. Animal experiments have confirmed that intramyocardial hydrogel injection has a significant inhibitory effect on left ventricular remodeling after MI, which is manifested by increasing scar thickness, inhibiting ventricular wall thinning and dilation, and improving cardiac function. Two days after injection of the hydrogel into the infarcted area, significant improvement in myocardial dyskinesia in the infarcted area was observed using 3D echocardiography (O'Connor et al., 2015). Computer models have also confirmed that injecting biomaterials can reduce wall stress and myocardial fiber stress and increase the ejection fraction; moreover, the distribution of biomaterials in the myocardial tissue is related to treatment efficacy at different pathological stages after MI.

Natural hydrogels extracted from organisms contain specific cell action sites, which have good cell affinity and biological effects such as promoting angiogenesis, promoting cell homing, and regulating inflammation after MI. Chitosan (Jayakumar et al., 2005), hyaluronic acid (Abdalla et al., 2013), ECM derivatives, and other natural hydrogels have been injected into the MI area. Experiments have confirmed that natural hydrogels can protect cardiac function after MI and inhibit left ventricular remodeling. However, natural hydrogels have limitations, such as limited sources, poor material repeatability, and difficult manual adjustment of the structure and mechanical properties.

Hao et al. introduced fullerenol nanoparticles with antioxidant effects into an alginate gel and found that the fullerenol/alginate gel can improve the survival of brown adipose stem cells (BASCs) in an ROS environment and their differentiation into cardiomyocytes (Hao et al., 2017). In a large clinical trial, 201 subjects received intracoronary IK-5001 injections, but the results showed that the application of the hydrogel did not reduce left ventricular remodeling and cardiac clinical events at 6 months, which differed from the results of preclinical animal experiments (Rao et al., 2016). These mixed results highlight the complexity of translating hydrogel therapy from bench to bedside. In animal experiments, chitosan hydrogels increased the thickness of the left ventricular wall and improved left ventricular remodeling and cardiac function. Additionally, chitosan-based hydrogels are easy to use in heart regeneration therapy because they encapsulate transplanted cells and bioactive molecules. Recently, Liu et al. found that chitosan hydrogels can improve the implantation rate of bone marrow mesenchymal stem cells (BMSCs) at the border of infarcted myocardium in mice, improve the ability of BMSCs to inhibit inflammatory reactions, and reduce the pyroptosis of vascular endothelial cells. In recent years, researchers have conjugated the conductive material polypyrrole with chitosan to prepare a conductive hydrogel (Liu et al., 2020). This hydrogel can synchronously contract isolated myocardial cell clusters *in vitro*, and *in vivo* experiments have confirmed that injecting the hydrogel into cardiac scar tissue can improve its electrical conductivity (Cui et al., 2018).

At present, myocardial ECM hydrogels are safe and effective for heart repair after MI (Wassenaar et al., 2016). Wassenaar et al. used transcriptome analysis to explore the mechanism of action of myocardial ECM hydrogels in preventing left ventricular remodeling and cardiac function decline after MI in rats (Wassenaar et al., 2016). They observed that the ECM hydrogel inhibited inflammatory responses, reduced cardiomyocyte apoptosis and hypertrophy, increased angiogenesis, promoted the expression of cardiac transcription factors, and recruited progenitor cells. Some researchers have added carbon nanotubes (CNT) with good conductivity and mechanical strength to collagen hydrogels and found that the addition of CNT can increase the elastic modulus of the gel to the level of adult rat ventricular muscle, while the conductivity is slightly higher than that of normal myocardium. The results of the co-culture of a CNT/collagen hydrogel and neonatal rat ventricular myocytes showed that the addition of CNT improved the adhesion, elongation, and contraction of cardiomyocytes (Sun et al., 2017). Awada et al. inserted TIMP-3 inhibitor, FGF-2, and SDF-1α into fibrin hydrogel, and directly injected the complex hydrogel into myocardium (Awada et al., 2017). Through the controlled release of the three complementary proteins, the cardiac function of MI rats receiving gel injection treatment was improved, matrix degradation and scar expansion were alleviated, and angiogenesis and stem cell recruitment were promoted.

Lee et al. developed an injectable sulfonated reversible thermal gel, SP-SHU-PNIPAM, generated from PNIPAAm-conjugated poly (hexamethylene serine urea) (PSHU) and sulfonate groups (Lee et al., 2019). The SPSHU-PNIPAM hydrogel injected into the myocardium improved cardiac function and angiogenesis after MI and reduced the infarct wall area and left heart wall thickness. In addition, using SPSHU PNIPAM to encapsulate VEGF reduced the initial burst of VEGF and promoted its sustained release. To improve the conductivity of the hydrogel, some researchers have chemically coupled gold nanoparticles to the PSHU-PNIPAAM lysine backbone to prepare a new conductive reversible thermal gel. Zhou et al. added graphene oxide nanoparticles to a biodegradable oligomeric (poly [ethylene glycol] fumarate) (OPF) hydrogel synthesized using PEG and fumaryl chloride. The introduction of graphene oxide improved the conductivity of the hydrogel and its adhesion to cells, promoted electrical signal transmission in the infarcted area and surrounding tissues of MI rats, upregulated the expression of gap junction proteins in myocardial cells, and improved cardiac function (Zhou et al., 2018). PEG-based hydrogels can be used as carriers for biomolecules and drugs. Carlini et al. developed a programmable cyclic peptide hydrogel containing a MMP/elastase digestion recognition sequence (Carlini et al., 2019). Under the action of MMP and elastase, it linearizes and quickly assembles into a gel, and the activation of the cyclic peptide and subsequent gel does not increase macrophage infiltration. Han et al. synthesized a PGN hydrogel using the peptides PA-GHRPS and NapFF and used it to encapsulate exosomes derived from human umbilical cord mesenchymal stem cells (UMSC Exo) (Han et al., 2019). They found that the exosomes were stably and continuously released into the PGN hydrogel (Wang et al., 2024). Injection of the UMSC Exo/PGN hydrogel around the infarct wall of MI rats reduced inflammatory reactions and fibrosis, improved cardiac function, and promoted angiogenesis. Koudstaal et al. used a urea pyrimidinone (UPy) hydrogel to carry insulin-like growth factor and hepatocyte growth factor and injected them into the myocardium of MI pigs (Koudstaal et al., 2014). Compared with a single injection of cytokines or hydrogels, they obtained a better cardiac protection effect. In addition, the number of endogenous myocardial stem/progenitor cells in the peripheral area of the infarction increased nearly four-fold compared to a single application of the hydrogel. Projahn et al. used synthetic hydrogels with different degradation rates to achieve time-sequential treatment of different substances after MI: N-terminal methylated chemokine (C-C motif) ligand 5 (Met-CCL5) carried by fast degradation hydrogels was released within 24 h after injection and inhibited early leukocyte infiltration; stromal cell-derived factor 1 (SDF-1) carried by slow degradation hydrogel was released in a gradient within 4 weeks after injection and recruited circulating hematopoietic stem cells to homing (Projahn et al., 2014).

Injectable hydrogels are reliable carriers for gene delivery and improve local targeting rates. Three types of target genes are available for the treatment of MI: those that improve the blood supply, those that alleviate fibrosis in the infarcted area, and those that regulates interactions between various factors. Li et al. used mesoporous silica nanoparticles (MSNs) as delivery carriers of miR-21-5p, encapsulated the MSNs/miR-21-5p complex into injectable hydrogels, and constructed a controlled release system for delivering miR-21-5p, which could reconstruct the function of macrophages, regulate the inflammatory microenvironment, and deliver miR-21-5p to endothelial cells to promote the formation of microvessels (Li et al., 2021). This study demonstrates the synergistic effect of the hydrogel and miR-21-5p in the treatment of MI. Small interfering RNA (siRNAs) have great potential for application in MI treatment. The siRNA can be efficiently transfected through the continuous and controllable release of the hydrogel into the local area.

### 4.2 Cardiac vascular regeneration

The human vascular system is responsible for delivering nutrients and oxygen to almost all organs and tissues, as well as for clearing the waste generated by organs. Hydrogels are unique biomaterials with high hydrophilicity, 3D network structure, swelling state, biological characteristics similar to the ECM, and self-support, allowing the diffusion and attachment of molecules and cells, playing an important role as carriers, ensuring the continuous local transmission of various therapeutic molecules, and becoming a fascinating therapeutic method in regenerative medicine. Chitosan is a polymer commonly used to prepare hydrogels and can be used in combination with anionic polymers. Sphingosine-1 phosphate (S1P) has a hydrophobic lipid tail and a negatively charged phosphate ester, which can promote the recruitment of endothelial progenitor cells to damaged or ischemic tissues and exert therapeutic angiogenesis effects. There are reports of the inhibitory effect of chitosan on angiogenesis (Shahzadi et al., 2020), and its regulatory effect on angiogenesis may be related to its relative molecular weight. However, these conflicting experimental conclusions require further investigation. The mechanism of angiogenesis is likely to involve multiple cells and signaling pathways, yet current applied research focuses only on 1 cell or signaling pathway. The targets of chitosan drug delivery systems are likely to be multifaceted. The vascularization strategy of tissue engineering is mainly achieved by directly loading cells with vascular differentiation potential and angiogenic cytokines or loading metal ions or drugs with pro-angiogenic properties into implants. Vascular regeneration factor such as VEGF, Alkaline fibroblast growth factor are most common used.

## 5 Role in biological pacing

Biological pacing seeks to restore physiological rhythm regulation using engineered cells or conductive materials as an alternative to electronic pacemakers. Hydrogels are pivotal in this field by: inducing spontaneous activity in cardiomyocytes; improving the electrode-tissue interface to enhance pacing efficiency; and acting as scaffolds for delivering pacemaker cells. In recent years, the use of stem cells, genes, and biomaterials for heart regeneration has become a popular research topic. Since the successful construction of the first tissue-engineered myocardium 20 years ago (LI et al., 2015) to the recent emergence of chambered cardiac organoids (mini-hearts) (Naumova and Iop, 2021), tremendous progress has attracted much attention. Strategies such as introducing pacing genes and cells into the heart, reprogramming the in-situ myocardium, and tissue engineering transplantation have been explored to find ideal solutions. These effects have been verified both *in vivo* and *in vitro* experiments (Naumova and Iop, 2021). In the above research, biodegradable materials have been widely and critically applied, which promote the development of biological pacing and conduction applications. The application of hydrogels in biological pacing is mainly reflected in three aspects: first, it can induce the spontaneous activity of myocardial cells to give them pacing characteristics; second, it optimizes the myocardial electrode interface of electronic pacemakers to improve pacing efficiency through anti-inflammatory, anti-fibrotic, and anti-conduction block effects; third, it serves as a scaffold and carrier for pacing cells, playing a role in constructing pacing tissues.

Regarding the spontaneous activity of cardiac myocytes induced by hydrogels, earlier studies induced cardiac myocytes to produce spontaneous activity by transfecting pacing genes and reprogramming. Recently, Hu et al. showed that a silk protein hydrogel could activate and transform rat ventricular myocytes into pacemaker cells, inducing the expression of cadherin glycoprotein in vascular endothelial cells (Hu et al., 2022). Although the low energy consumption of pacemakers and improvements in battery technology continues, re-performing pacemaker transplantation owing to battery life and endurance ability remains an unsolved problem. Therefore, improving the contact interface between the myocardial tissue and electrodes can lower the pacing voltage threshold, which can improve the efficiency of pacemakers and extend battery life. For this reason, Zhao et al. tested a type of biomaterial called PAMB-G hydrogel, which was proven to be able to conduct efficient electrical conduction through isolated myocardial experiments and achieved excellent performance in terms of conduction velocity and field potential amplitude. Hydrogels can be used as carriers for pacemaker cells that can be injected into the heart. They can also serve as scaffolds for pacemaker cells, forming a pacemaker cell scaffold complex *in vitro*, which can be further cultured into pacemaker tissue and transplanted into the heart. Tao et al. planted cardiac cells from neonatal rats onto fibrin gels to form spontaneously contractile heart tissue sheets *in vitro* (Tao et al., 2017). Zhang et al. used Matrigel matrix gel to create a composite with pacemaker cells derived from progenitor cells, cultured them *in vitro* to form pacemaker tissue, transplanted them into animal ventricles, and confirmed the production of functional ectopic pacemaker sites *in vivo* (Zhang et al., 2017). Zhou et al. introduced graphene oxide nanoparticles into the hydrogel to prepare a conductive hydrogel called OPF/GO. After injection into the heart, it was confirmed that the expression of relevant gap junction proteins could be upregulated through the Wnt signaling pathway, improving the electrical conduction between the normal myocardium and the scarred myocardium, and had an obvious role in maintaining cardiac function after MI. Zyl et al. used nano cellulose carbon nanotubes to form conductive hydrogels and injected them into a Langendorff-isolated heart after radiofrequency ablation to produce a myocardial block, which confirmed that the prepared hydrogel could restore the electrical conduction of the blocked myocardium (Zyl et al., 2020). Zhang et al. polymerized 3-amino-4-methoxybenzoic acid (AMB) with ammonium persulfate to form the polymer PAMB and crosslinked gelatin with N - (3-dimethylaminopropyl)-N′-ethylcarbodiimide hydrochloride and N-hydroxysuccinimide to prepare the crosslinked conductive hydrogel PAMB-G. PAMB-G was injected into the scar tissue of rats with MI and confirmed to improve electrical impulse conduction in the damaged area, maintain normal ventricular function, and reduce arrhythmia (Zhang et al., 2020). Fu et al. also injected PAMB-G into the infarcted myocardial area of rats, further confirming the long-term (1 year) effect of the conductive hydrogel on cardiac conduction, indicating that it is non-toxic for a long time and plays a role in maintaining cardiac function (Fu et al., 2022).

## 6 Use in heart failure

Chronic heart failure (CHF) has a high incidence and mortality rate worldwide. In recent years, the development of new drugs and innovative breakthroughs in biological tissue engineering technologies are expected to improve the treatment of this group of patients. Alginate hydrogels, a new type of biomaterial, have been used in animal experiments and clinical trials for heart failure and have achieved ideal therapeutic effects.

At present, there are two main types of alginate hydrogels used in animal research and clinical trials: Algisyl LVR and IK 5001 (formerly BL-1040). The former was formed by dissolving sodium alginate and calcium alginate in a 4.6% sterile mannitol solution, and its gelation process relied on a local high-calcium environment formed by mixing the two components. After injection into animals, Algisyl LVR gradually degraded with a decrease in calcium ion concentration. IK 5001 is a mixture of 2% sodium alginate and 0.6% calcium gluconate. It can be administered via intracoronary or intramyocardial injection. They can be gelled in the high-calcium environment formed by MI. With the fibrosis of the infarct, the concentration of calcium ions gradually decreases, and the gel gradually degrades.

### 6.1 BL-1040

Leor et al. (2009) found that when BL-1040 was injected into the injured blood vessels of pigs with MI, the hydrogel was extravasated to the lesion site of the infarct-related vessels owing to its high permeability. It solidified in the high-calcium environment generated by tissue necrosis and was gradually replaced by myocardial fibroblasts and collagen fibers, which increased the thickness of the tissue at the lesion site and significantly reduced the end-diastolic and end-systolic volumes of the left ventricle, thereby improving the filling capacity of the ventricle. The study also found that the 2 mL and 4 mL alginate hydrogels had similar efficacies and were better than the 1 mL dose group. Compared with the control group, there was no significant increase in cardiac arrhythmia, myocardial enzymology changes, or other events in the treatment group during the entire observation period. The forward blood flow of the coronary artery and myocardial microcirculation were not affected, and there was no distal organ embolism such as in the liver, spleen, or kidney, suggesting that the appropriate BL-1040 hydrogel injected into the coronary artery had good feasibility, safety, and effectiveness. This hydrogel could repair the MI area, reverse ventricular remodeling, and prevent the progression of heart failure. In the early stage of MI and 2 months after MI, injection of a hydrogel into the infarct can increase the thickness of scars and improve left ventricular systolic and diastolic dysfunction; notably, the effect of early treatment is not inferior to or even better than that of neonatal cardiomyocyte transplantation. It can be seen from the above studies that, whether administered by intracoronary injection or local myocardial injection, alginate saline gel can delay the process of heart failure after MI.

### 6.2 Algisyl LVR hydrogel

Sabbah et al. (2013) used a dog model of CHF to study an Algisyl LVR hydrogel. After thoracotomy, seven sites with a spacing of 1.0–1.5 cm were selected along the ventricular free wall from the anterior to the posterior interventricular sulcus, from the apex to the bottom of the heart as injection targets. 0.25–0.35 mL Algisyl LVR hydrogel was injected at each point, and the control group was administered normal saline. The results showed that intramyocardial injection of the drugs was safe. Although there may be a small number of premature ventricular beats in the early stage after injection, they can improve on their own within 10–15 min without the need for drug intervention. During the 17-week observation period, there were no sudden deaths or use of vasoactive drugs. Hydrogel treatment can thicken the left ventricular wall at the end of the systolic and diastolic phases, improve the slope of the left ventricular pressure-volume relationship curve at the end of the systolic phase, reduce the proportion of severe mitral regurgitation and volume at the end of the systolic and diastolic phases, and significantly increase the left ventricular ejection fraction (LVEF), end systolic sphericity index (ESSI), and E-peak deceleration time. Among them, the LVEF of the Algisyl LVR group significantly increased from 26% ± 0.4% to 31% ± 0.4%, while the control group significantly decreased from 27% ± 0.3% to 24% ± 1.3%, (P < 0.05). Sabbah et al. (2013) continued to use the above animal models to study the early efficacy of Algisyl LVR hydrogel. The results showed that the left ventricular end diastolic volume [(63 ± 2.4 mL vs. (69 ± 2.3) mL] and end systolic volume [(39 ± 1.3) mL vs. (50 ± 1.8) mL] in dogs were significantly smaller after 3 h of hydrogel injection, while LVEF [(38 ± 1.2)% vs. (28 ± 0.8)%], ESSI [(1.45 ± 0.06) vs. (1.27 ± 0.08)] and the thickness of the left ventricular end systolic front and rear walls [(0.92 ± 0.02) cm vs. (0.80 ± 0.02) cm, (0.93 ± 0.01) cm vs. (0.80 ± 0.01) cm] were all increased. Hydrogel injections can improve cardiac function during the early stages.

## 7 Translational challenges

Although early studies demonstrated the safety and potential promise of intracoronary alginate hydrogel delivery, the neutral results from pivotal clinical trials indicate that its definitive therapeutic benefits and clinical application value require further investigation. The cases of IK-5001 and Algisyl LVR collectively illustrate that the successful translation of hydrogel therapy from preclinical research to clinical application remains challenging, necessitating a deeper understanding of its mechanisms of action and the optimization of patient selection strategies.

For a scaffold to be clinically translatable, crucial factors such as safety, biocompatibility, reproducible and scalable manufacturing, and minimally invasive deliverability must also be considered. Future research should also focus on smart hydrogels that actively interact with the post-MI microenvironment: enzyme-responsive systems for spatially/temporally controlled release; pH-responsive systems that activate in the acidic infarct zone; refined temperature-responsive systems for optimal gelation; and ultimately, multi-responsive platforms for intelligent, feedback-driven therapy.

## 8 Conclusion and future directions

Recently, hydrogels have been extensively studied in heart tissue engineering. Injectable hydrogels have been proven suitable for MI, and some hydrogels have been used in clinical trials. Hydrogels can be used as carriers to deliver cells, bioactive molecules, and drugs to infarcted hearts, improve the survival rate of transplanted cells, and compensate for myocardial cell loss and damage to cardiac function caused by MI. Among all biomaterials used for heart tissue engineering, hydrogels not only provide mechanical support to the heart but also serve as carriers of cells, growth factors, and drugs, owing to their characteristics of being minimally invasive, controllable, having slow delivery, and their *in vivo* decomposition into harmless derivatives. These characteristics demonstrate their great potential for application in the repair and regeneration of damaged myocardium after MI. Accelerating the clinical translation of cardiac hydrogels requires a concerted focus on several fronts: prioritizing large-animal models for clinically predictive validation; adopting standardized reporting criteria to enhance reproducibility; conducting long-term safety studies to assess biocompatibility and degradation; and evaluating sex-specific efficacy to ensure broad applicability.

However, the transition from scientific research to clinical practice remains challenging. Although injectable hydrogels are promising for the clinical treatment of cardiovascular diseases, their biodegradation mechanism *in vivo* is still not fully understood, and many problems remain to be solved. For example, because of the biological differences between experimental animals and humans, it is unclear whether the injection time, dosage, pathway, and degradation time of hydrogels can be controlled. Similarly, the choice of degradation time also needs to be considered. Further research on the impact of degradation products and the selection of gel materials is also required.

## Abbreviations

ECM, extracellular matrix; SEM, scanning electron microscope; TEM, transmission electron microscope; DECM, decellularized extracellular matrix; GelMA, gelatin methacryloyl; HA, hyaluronic acid; iPS-CMs, induced pluripotent stem cell differentiated cardiomyocytes; PNIPAAm, poly N-isopropylacrylamide; PEG, polyethylene glycol; MI, myocardial infarction; MVO2, myocardial oxygen consumption; NT pro BNP: N-terminal pro-B-type natriuretic peptide; b-FGF, basic fibroblast growth factor; VEGF, vascular endothelial growth factor; BMSC, bone marrow mesenchymal stem cells; CNT, carbon nanotubes; DPC, dihydro-4-one-3-carboxylic acid; MP, matrix metalloproteinase.
